# From Sleep Tracking to Early Parkinson’s Disease Diagnosis: A Promising New Frontier

**DOI:** 10.31662/jmaj.2024-0193

**Published:** 2024-10-08

**Authors:** Liang-Kung Chen

**Affiliations:** 1 Center for Geriatrics and Gerontology, Taipei Veterans General Hospital, Taipei, Taiwan; 2 Center for Healthy Longevity and Aging Sciences, National Yang Ming Chiao Tung University, Taipei, Taiwan; 3 Taipei Municipal Gan-Dau Hospital (Managed by Taipei Veterans General Hospital), Taipei, Taiwan

**Keywords:** Aging, cognition, depression, exercise, physical activity

Driven by the potential for improved patient outcomes, early detection of Parkinson’s disease (PD) and associated conditions remains a paramount goal for clinicians and researchers ^(1)^. A new study by Tanaka et al. sheds light on a simple yet potentially important indicator of PD and related disorders, i.e., the number of times a person turns over during sleep. In the comparative polysomnographic study involving 33 persons with PD and 57 healthy controls, nocturnal turning was significantly reduced in the PD group ^(2)^. This measure emerged as the only independent factor associated with PD in multivariate analysis, outweighing other variables such as age, muscle mass, and strength. The authors suggested that reduced sleep activity, specifically turning frequency during sleep, could be an early indicator of PD and related disorders, potentially preceding established motor symptoms, with significance for diagnosis and understanding disease progression. One of the most intriguing aspects of the study is the identification of specific cutoff values for the number of nocturnal turns that fewer than 6 turns per night were associated with PD. Moreover, the authors also found sex-specific cutoffs―fewer than 9 turns for men and fewer than 6 turns for women. These thresholds demonstrated high sensitivity and specificity, suggesting potential as a screening tool. This underscores the importance of considering sex-specific norms when evaluating sleep activity. Although promising, this study has limitations that warrant consideration. The sample size was relatively small, and the patient group had mostly early-stage disease. Furthermore, the use of visual observation to count turns, while practical, may be less precise than accelerometer-based measurements. In addition to the abovementioned limitations, the study also leaves some questions unanswered. For instance, what drives the reduction in turning frequency in people with PD? Is it primarily a motor symptom, or do other factors, such as sleep architecture changes or autonomic dysfunction, play a role? To establish a causal link between reduced nocturnal turning and PD, longitudinal studies with larger sample sizes are needed.

Despite these limitations, this study opens exciting avenues for both clinical practice and future studies. If validated in larger cohorts, nocturnal turning frequency could become a valuable addition to the toolkit for early PD detection. Its noninvasive nature and ubiquity of sleep monitoring technology make it an attractive option for widespread screening. Moreover, this study highlights the potential of examining subtle motor changes during sleep as a window into neurodegenerative processes ^(3)^. Sleep disturbances are a well-known nonmotor symptom of PD; however, most research has focused on issues such as REM sleep behavior disorder or insomnia. While PD is associated with a variety of sleep disturbances, such as insomnia, excessive daytime sleepiness, and REM sleep behavior disorder, accurate diagnosis of these sleep disorders in people with PD can be challenging due to overlapping symptoms and the influence of the disease itself ^(4)^. REM sleep behavior disorder (RBD) is a precursor to PD, affecting over half of its patients. Neuroimaging studies reveal alterations in dopamine, acetylcholine, and brain structure and function beyond the brainstem in both RBD and PD, suggesting RBD as a multisystem neurodegenerative process ^(5)^. By zeroing in on the quantitative aspects of sleep movements, this work offers a fresh perspective on how PD affects motor function during sleep. These findings also have implications for understanding the trajectory of motor symptom development in PD. The fact that reduced turning frequency may precede clinically apparent muscle weakness suggests that subtle motor changes may occur earlier in the disease course than was previously recognized. This information could inform both our models of disease progression and strategies for early intervention. From a practical standpoint, the identification of specific cutoff values for nocturnal turns provides clinicians with a concrete metric to consider when evaluating patients. While further validation is needed before this could be used as a standalone screening tool, it could be a valuable addition to the constellation of early signs that raise suspicion for PD.

Looking ahead, this study suggests some promising research directions. Longitudinal studies tracking changes in sleep activity from prodromal to advanced stages of PD could provide insights into how this marker evolves over time. Combining sleep movement data with other early indicators, such as olfactory dysfunction or subtle cognitive changes, could yield more powerful predictive models for PD risk. There is also potential for leveraging this finding in intervention studies. In conclusion, while further research is needed to fully validate and contextualize these findings, this study represents an important step forward in our quest for early PD detection. By shedding light on the subtle motor changes occurring during sleep, it offers both a potential new screening tool and fresh insights into the progression of motor symptoms in PD. As we continue to unravel the complex tapestry of PD pathology, every new thread of understanding brings us closer to better diagnosis, treatment, and, ultimately, prevention of this challenging disease. The simple act of turning over in bed may speak volumes about neurological health.

## Article Information

### Conflicts of Interest

None

### Disclaimer

Liang-Kung Chen is one of the Editors of JMA Journal and on the journal’s Editorial Staff. He was not involved in the editorial evaluation or decision to accept this article for publication at all.
